# RNA Helicase A/DHX9 Forms Unique Cytoplasmic Antiviral Granules That Restrict Oncolytic Myxoma Virus Replication in Human Cancer Cells

**DOI:** 10.1128/JVI.00151-21

**Published:** 2021-06-24

**Authors:** Masmudur M. Rahman, Ami D. Gutierrez-Jensen, Honor L. Glenn, Mario Abrantes, Nissin Moussatche, Grant McFadden

**Affiliations:** a Center for Immunotherapy, Vaccines, and Virotherapy Biodesign Institute, Arizona State University, Tempe, Arizona, USA; b Department of Molecular Genetics and Microbiology, University of Florida, Gainesville, Florida, USA; University of Illinois at Urbana Champaign

**Keywords:** DHX9, myxoma virus, RNA helicase A, antiviral granules, human cancer cells, oncolytic viruses, poxvirus, virus-host interactions

## Abstract

RNA helicase A/DHX9 is required for diverse RNA-related essential cellular functions and antiviral responses and is hijacked by RNA viruses to support their replication. Here, we show that during the late replication stage in human cancer cells of myxoma virus (MYXV), a member of the double-stranded DNA (dsDNA) poxvirus family that is being developed as an oncolytic virus, DHX9, forms unique granular cytoplasmic structures, which we named “DHX9 antiviral granules.” These DHX9 antiviral granules are not formed if MYXV DNA replication and/or late protein synthesis is blocked. When formed, DHX9 antiviral granules significantly reduced nascent protein synthesis in the MYXV-infected cancer cells. MYXV late gene transcription and translation were also significantly compromised, particularly in nonpermissive or semipermissive human cancer cells where MYXV replication is partly or completely restricted. Directed knockdown of DHX9 significantly enhanced viral late protein synthesis and progeny virus formation in normally restrictive cancer cells. We further demonstrate that DHX9 is not a component of the canonical cellular stress granules. DHX9 antiviral granules are induced by MYXV, and other poxviruses, in human cells and are associated with other known cellular components of stress granules, dsRNA and virus encoded dsRNA-binding protein M029, a known interactor with DHX9. Thus, DHX9 antiviral granules function by hijacking poxviral elements needed for the cytoplasmic viral replication factories. These results demonstrate a novel antiviral function for DHX9 that is recruited from the nucleus into the cytoplasm, and this step can be exploited to enhance oncolytic virotherapy against the subset of human cancer cells that normally restrict MYXV.

**IMPORTANCE** The cellular DHX9 has both proviral and antiviral roles against diverse RNA and DNA viruses. In this article, we demonstrate that DHX9 can form unique antiviral granules in the cytoplasm during myxoma virus (MYXV) replication in human cancer cells. These antiviral granules sequester viral proteins and reduce viral late protein synthesis and thus regulate MYXV, and other poxviruses, that replicate in the cytoplasm. In addition, we show that in the absence of DHX9, the formation of DHX9 antiviral granules can be inhibited, which significantly enhanced oncolytic MYXV replication in human cancer cell lines where the virus is normally restricted. Our results also show that DHX9 antiviral granules are formed after viral infection but not by common nonviral cellular stress inducers. Thus, our study suggests that DHX9 has antiviral activity in human cancer cells, and this pathway can be targeted for enhanced activity of oncolytic poxviruses against even restrictive cancer cells.

## INTRODUCTION

Large DNA viruses, such as members of the poxvirus family, encode dozens of immune-modulatory proteins that can deflect or evade host immune responses to enable successful viral replication and pathogenesis. However, depending on the evolutionary history of the specific poxvirus, many of these host interactive viral proteins often function in a highly species-specific fashion which can cause the virus to be permissive only in a subset of host species. This unique virus-specific host restriction has allowed the exploitation of certain poxviruses for use as vaccine vectors, gene delivery vehicles, or oncolytic viruses for the treatment of cancer. Myxoma virus (MYXV) is the prototypic member of the *Leporipoxvirus* genus of the Poxviridae family, which causes a lethal disease called myxomatosis only in European rabbits (Oryctolagus cuniculus) but is completely nonpathogenic for all other nonleporid host species. MYXV is currently being developed as an oncolytic virus for human cancer, because of its safety in humans combined with an ability to productively infect many (but not all) classes of cancer cells both *in vitro* and *in vivo* within tumor tissues (1). The unique permissiveness of most mouse or human cancer cells to MYXV infection is mainly because these cells either lost or have inactivated elements of their innate antiviral responses to the virus infection. For example, the antiviral pathways induced by type I interferon (IFN) and tumor necrosis factor (TNF), two antiviral cytokines that can restrict MYXV replication in normal human or mouse cells, are frequently defective in many cancer cells (2–5). Although MYXV can infect the vast majority of transformed or cancer cells tested to date, this productive replication largely relies on whether the virus is able to successfully overcome the diverse antiviral signaling pathways still active in these cancer cells (6). In addition, viruses also modulate metabolic pathways in highly proliferative cancer cells. Many of the host pathways or molecules that can functionally restrict virus replication in cancer cells are yet to be identified for development of more universally effective oncolytic viruses. In case of MYXV, the known cellular pathways that govern MYXV tropism in cancer cells are (i) endogenously activated protein kinase B (PKB)/AKT, (ii) cellular tumor suppressors such as p53, ataxia-telangiectasia mutated (ATM), and retinoblastoma (Rb), (iii) the antiviral pathways activated by protein kinase R (PKR), (iv) antiviral states induced by interferons or TNF, and (v) recently identified members of the cellular DEAD box RNA helicase family (7–11).

DExD/H-box (DEAD box) RNA helicases play essential roles in all cellular processes where RNAs are involved (12). In addition, several family members are known to be hijacked and regulated by viral proteins, for example those involved in viral RNA processing or antiviral host defenses (13). Among the RNA helicases, DHX9, also known as Nuclear DNA helicase II (NDH II) or RNA helicase A (RHA), contains the DExH-box (DHX) and is an NTP-dependent helicase capable of unwinding both double-stranded RNA (dsRNA) and dsDNA (14). DHX9 is a large multidomain and multifunctional protein, critically involved in DNA replication, transcription, translation, RNA processing and transport, maintenance of genomic stability, and many other functions. DHX9 is predominantly localized in the nucleus but is able to shuttle to the cytoplasm to engage in some of its functions. This DHX9 nuclear shuttling is dependent on the presence of a nuclear localization signal (NLS) and a nuclear export signal (NES), both of which are present at the C-terminal region (15). DHX9 is also required for optimal replication of many RNA and DNA viruses, including HIV-1 (16–19), hepatitis C Virus (20), influenza A (21), hepatitis E (22), cytomegalovirus (23), adenovirus (24), classical swine fever (25), and foot and mouth disease viruses (26). For many of these viruses, DHX9 has been identified as a cellular binding partner for many key virus-encoded proteins that regulate virus tropism.

Although required for optimal replication of many viruses that modulate DHX9, it also has the potential to sense viral nucleic acids and trigger the activation of antiviral immunity. This suggests that there is a dynamic battle between virus and host cells to control DHX9 functions. DHX9 has been identified as a CpG DNA-binding protein in plasmacytoid dendritic cells (pDCs), which allow induction of cytokine responses via interaction with MyD88 (27). DHX9 also interacts with viral RNA in myeloid DCs, and in these cells it activates antiviral responses by signaling through mitochondrial antiviral-signaling protein (MAVS) (28). A recent study reported that DHX9 functions with a newly identified inflammasome sensor, Nlrp9b, in intestinal epithelial cells to recognize dsRNA from rotavirus (29). DHX9 is also involved in innate immune responses against DNA viruses in different primary or immune cell types. This immune response is mainly mediated by the nuclear DHX9, where it is involved in the transcriptional regulation of genes involved in innate immunity (30, 31).

Due to the complex nature of DHX9 functions, it has also been implicated for both oncogenesis and tumor-suppressive properties. However, the role(s) ascribed to DHX9 depends on the cellular context, its interacting partners, and in some cases, even the levels of expressed protein. For example, analyses of lung cancer samples consistently identified DHX9 overexpression compared to normal lung tissues (32). DHX9 levels and interactions with oncogenes have been implicated in other types of cancers, such as breast and ovarian cancer, osteosarcoma, and Wilms’ tumor (33, 34). These findings collectively suggest that success with therapeutic oncolytic viruses might be closely linked with the management of DHX9 protein and functions in cancer cell targets.

Our recent report on the screening of the cellular RNA helicase superfamily identified several RNA helicases that have potential antiviral and/or proviral functions in human cancer cells for the replication of oncolytic MYXV (11). DHX9 was identified as one of the antiviral RNA helicases for which targeted knockdown enhanced MYXV replication in multiple cancer cell types that are normally permissive, semipermissive, and nonpermissive to MYXV replication. In certain members of the cancer cell types tested, knockdown of endogenous levels of DHX9 using RNAi significantly enhanced MYXV replication. Here, we report a novel antiviral function of DHX9, where it forms “antiviral granules” in the cytoplasm that can restrict MYXV in at least some human cancer cells by sequestering essential viral factors and thus restricting late stages of the replication cycle.

## RESULTS

### MYXV infection activates the formation of cytoplasmic DHX9 antiviral granules.

**FIG 1:**
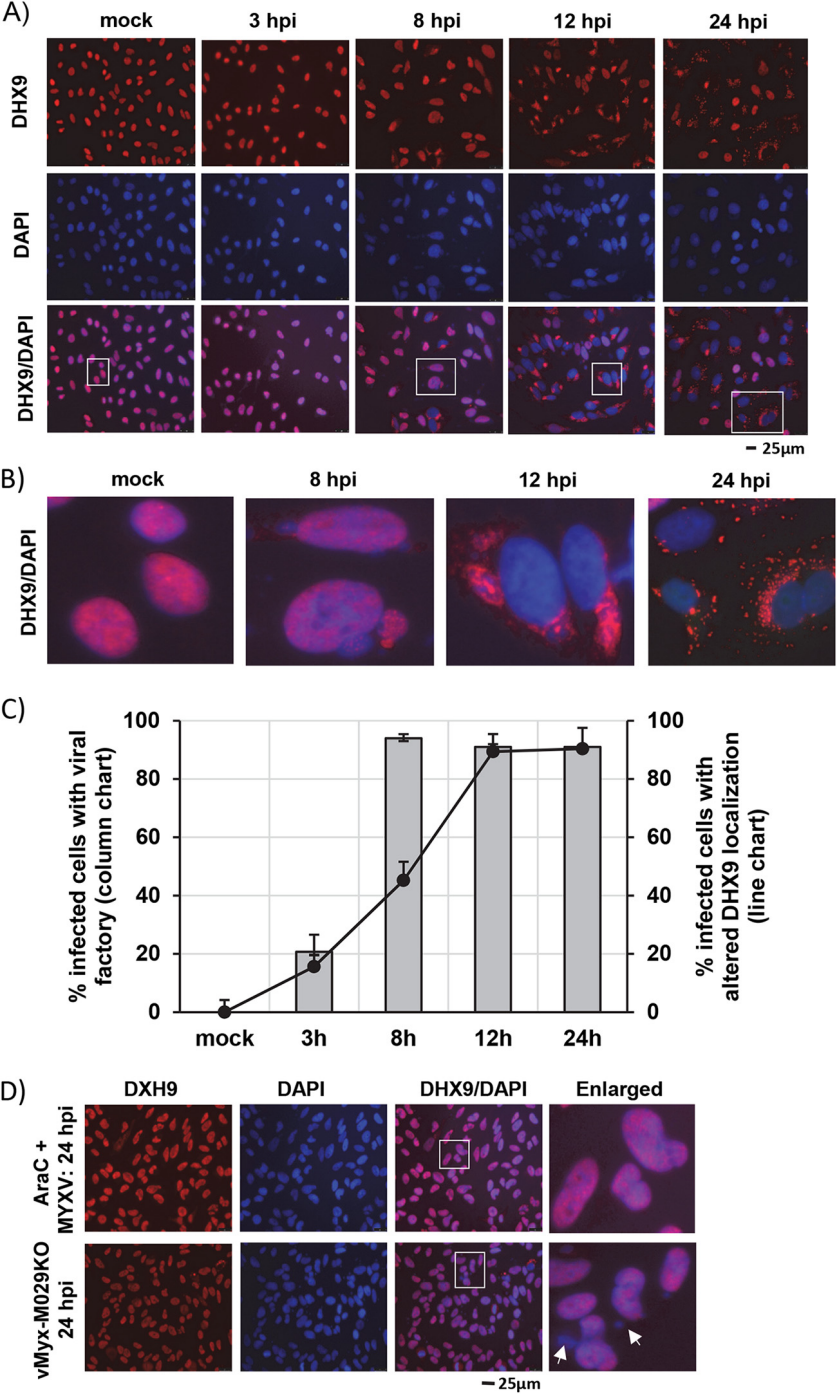
DHX9 forms cytoplasmic antiviral granules after MYXV infection. (A) A549 cells were seeded on glass-bottom 35-mm dishes and left overnight to adhere. The next day, cells were mock-infected or infected with WT-MYXV (MOI = 1.0). At the indicated time points, cells were fixed and stained with antibodies against DHX9. Nuclei were stained with DAPI. (B) Images are enlarged from the bottom panel of A. (C) Number of cells showing viral factory and altered nuclear localization of DHX9 at different time points after infection. A minimum of 100 cells were used for analysis from samples in panel A using ImageJ. (D) A549 cells were treated with AraC for 30 min before infection with WT-MYXV (MOI = 1.0) for 24 h (top panel) or infected with vMyx-M029KO (MOI = 5.0) virus for 24 h, and the cells were fixed and stained with antibodies and imaged with a fluorescence microscope. Viral factories stained with DAPI are indicated with arrows.

MYXV, like all poxviruses, replicates exclusively in the cytoplasm of infected cells and requires relatively little from the host cell nucleus to complete the viral replication cycle. Previously, we reported that, in certain human cancer cells, knockdown of DHX9 using RNAi significantly enhanced MYXV replication, suggesting that DHX9 is capable of mediating antiviral functions against MYXV (11). To understand how DHX9 functions to enact an antiviral role against MYXV, we studied the cellular localization of DHX9 using immunofluorescence (IF) microscopy during the MYXV replication cycle. Previous reports suggest that DHX9 is predominantly localized in the nucleus (30, 35, 36). We also observed that in uninfected human A549 lung carcinoma cells and other human cancer cell lines, DHX9 is mostly localized in the nucleus as observed using IF (Fig. 1A and data not shown). But after MYXV infection, the localization of DHX9 is altered during the progression of the virus replication cycle. At the late replication cycle (12 hours postinfection [hpi] and 18 hpi), DHX9 stained as punctate granular structures in the cytoplasm, which in some cases were associated with viral factories (Fig. 1A and B). These granular structures will be called “DHX9 antiviral granules” here. We also quantified the percentage of infected cells with altered localization of DHX9, based on the formation of viral factory and DHX9 staining, which suggests that more than 90% of infected cells at 24 hpi had altered DHX9 localization and formation of antiviral granules (Fig. 1C). In order to better understand whether the formation of DHX9 antiviral granules is dependent on the specific stages of the viral replication cycle, we used AraC, which blocks poxvirus DNA replication and late protein synthesis. As expected, treatment of cells with AraC before infection with MYXV completely inhibited formation of viral factories in the cytoplasm and late viral protein synthesis. In these cells when DHX9 localization was studied using IF, DHX9 was detected only in the nucleus even after 24 hpi (Fig. 1D, top panels). We next examined DHX9 localization and formation of antiviral granules using vMyx-M029KO virus, which is defective in late protein synthesis and progeny virus formation in all human cells tested in the absence of M029 expression (9). M029-KO virus infection in human cells does result in early gene expression and DNA replication, allowing formation of small viral factories. When A549 cells were infected with M029-KO virus, after 24 hpi we observed that the majority of cells (∼90%) had DHX9 signal in the nucleus, and in some cells both nucleus and viral factories, but rarely was there any visualization of DHX9-positive antiviral granules (Fig. 1D, bottom panels). Together, these results suggest that DHX9 formation of antiviral granules depends on the progression to the late replication cycle of MYXV in human cells.

**FIG 2:**
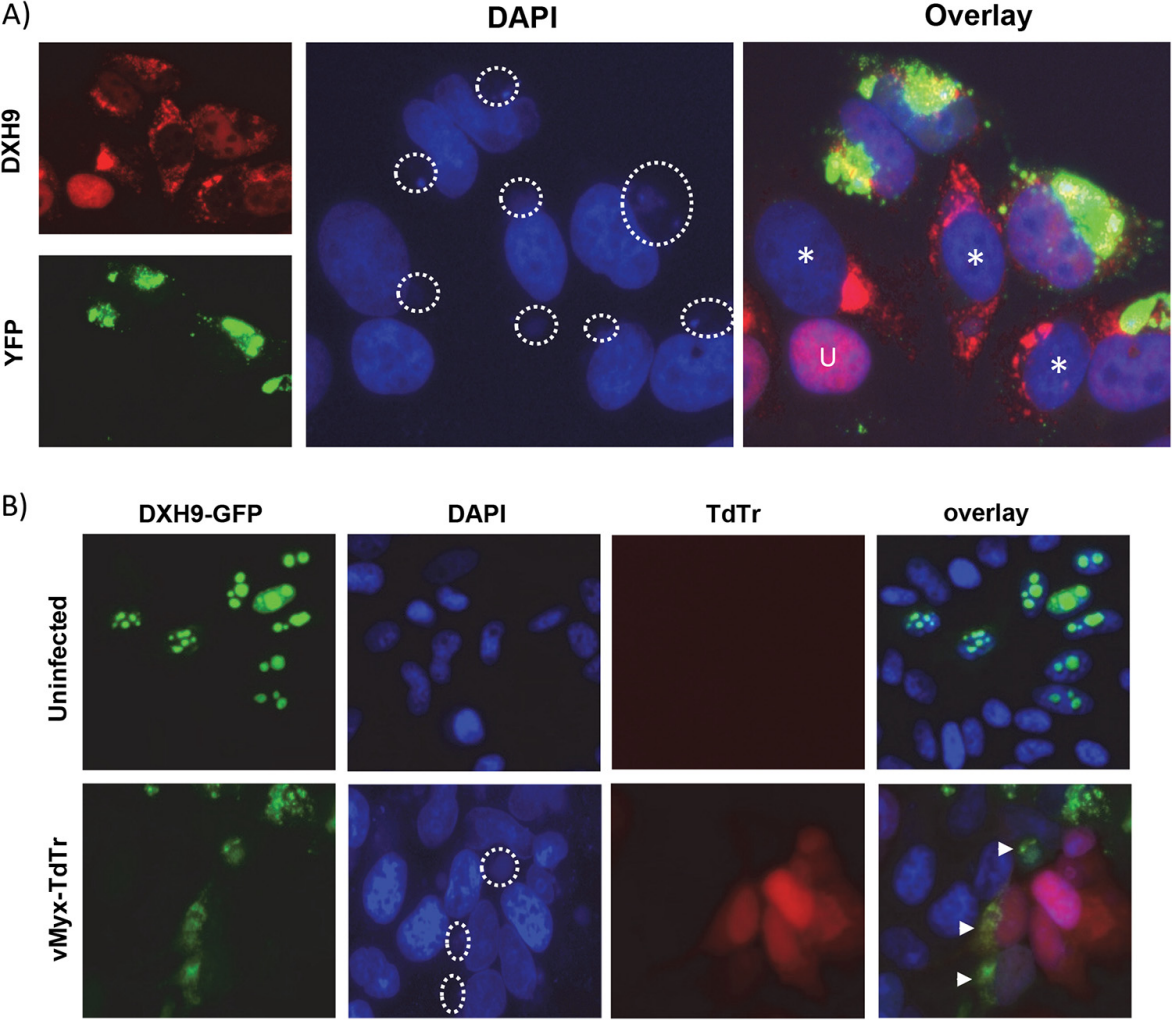
DHX9 localization is altered during MYXV infection and forms antiviral granules. (A) A549 cells were infected with vMyx-Venus/M093 (MOI = 1.0). After 24 hpi, cells were fixed and stained with antibodies against DHX9. Nuclei were stained with DAPI. (B) A plasmid expressing DHX9-GFP fusion protein was transfected in HeLa cells seeded on glass-bottom 35-mm dishes. After 48 h posttransfection, cells were mock-infected or infected with wild-type vMyx-TdTr (MOI = 1.0). After 24 hpi, cells were fixed and stained with DAPI, and images were taken using a fluorescence microscope. Viral factories in the infected cells are indicated with circles in the DAPI panels; U, uninfected cells; cells without progeny virus formation but altered DHX9 localization are indicated (*). Cells with altered DHX9-GFP localization are indicated with arrows.

To further understand at which stage of virus replication DHX9 localization is altered and forms antiviral granules, we infected A549 cells with MYXV-forming reporter protein Venus-labeled virions during the replication cycle. Using this virus, we can demonstrate that DHX9 localization is altered in the cells with a viral factory and before mass production of progeny virions (Fig. 2A). To further confirm that MYXV infection altered the localization of DHX9, we used a plasmid expressing green fluorescent protein (GFP)-tagged DHX9 (36). As reported before, in transfected HeLa cells, we observed localization of DHX9-GFP in the nucleus. However, when the cells were infected with vMyx-TdTr (a recombinant MYXV expressing reporter protein Tdtomato red under a poxvirus synthetic early/late promoter), the DHX9-GFP protein signals were observed in the cytoplasm of the infected cells (Fig. 2B). Apart from MYXV, infection of A549 cells with either VACV or CPXV also caused cytoplasmic localization of DHX9 during the late replication cycle (data not shown).

### DHX9 regulates MYXV late stages of replication in all types of human cancer cells.

**FIG 3:**
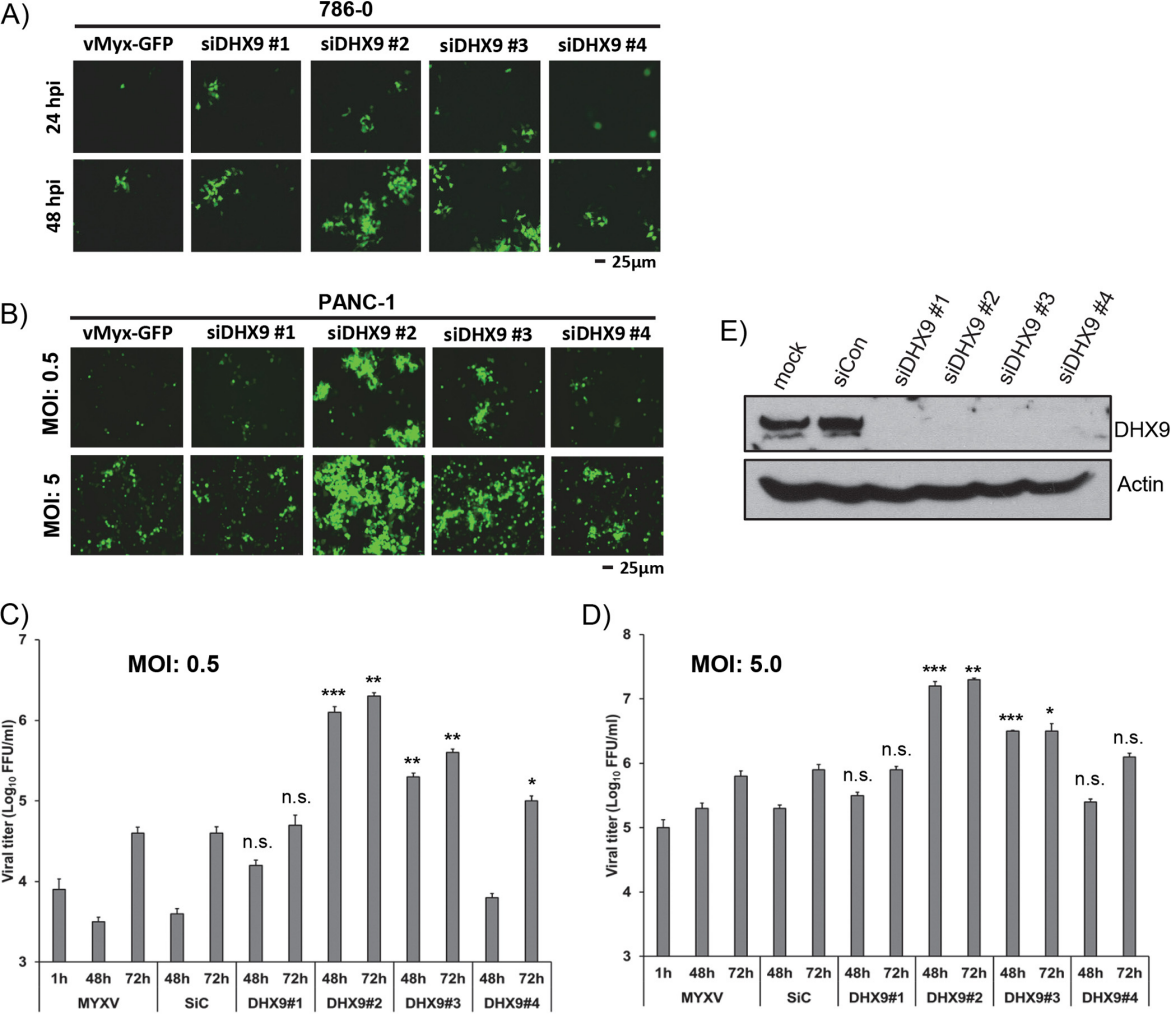
DHX9 knockdown enhances MYXV replication in human cancer cells. (A) 786-0 cells were transiently transfected with individual siRNAs targeted to DHX9. After 48 h, the cells were infected with vMyx-GFP (MOI = 0.01) for 1 h and replaced with fresh medium. After 24 h, live images were captured from each well using an Evos microscope. Shown here are images at 24 and 48 hpi of the same foci. (B to D) PANC-1 cells were transiently transfected with individual DHX9 siRNAs. After 48 h, the cells were infected with vMyx-GFP at an MOI of 0.5 or 5.0 for 1 h and replaced with fresh medium. Images showing expression of GFP after 3 days postinfection (B). The cells were harvested at 48 or 72 hpi to determine progeny virus formation by titration assay on permissive RK13 cells. The virus titers were determined in triplicate. Virus titers after infection with an MOI of (C) 0.5 or (D) 5.0. Statistically significant differences in comparison to infection with MYXV alone or cells transfected with control siRNA (SiC) are indicated; *, *P* < 0.05; **, *P* < 0.01; ***, *P* < 0.001; ns, not significant. (E) Western blot analysis of DHX9 protein level in PANC-1 cells after transfection of individual siRNA; actin as loading control.

**FIG 4:**
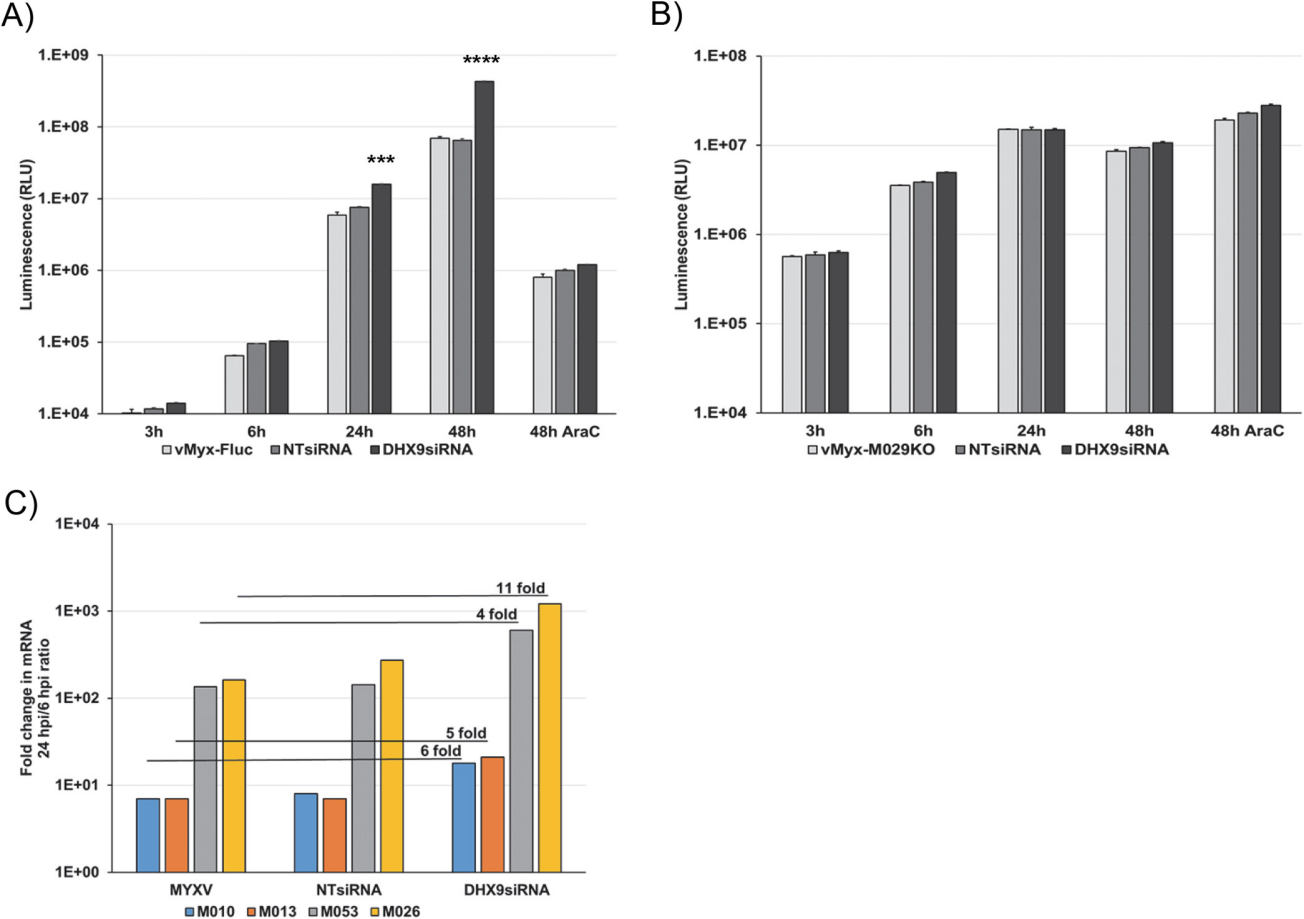
Knockdown of DHX9 enhances the expression of MYXV late mRNA and proteins. A549 cells were transfected with control NTsiRNA or DHX9 siRNA no. 2. (A and B) After 48 h, the cells were infected with (A) vMyx-FLuc (MOI = 0.1) or (B) vMyx-M029KO-FLuc (MOI = 1.0) for 1 h and replaced with fresh medium. Cells were then collected at the indicated time points and processed for the luciferase assay. The assays were done in triplicate. Statistically significant differences in comparison to infection with MYXV alone are indicated; ***, *P* < 0.001; ****, *P* < 0.0001. (C) Relative quantification of selected MYXV genes expression after siRNA transfection. The values in fold changes are based on comparing MYXV infection without and with DHX9 siRNA transfection.

We next studied the impact of DHX9 antiviral granules on MYXV replication in human cancer cells. We previously reported that in certain types of human cancer cell lines (particularly those that are naturally semipermissive or nonpermissive for MYXV), DHX9 knockdown enhanced progeny virus formation (11). Even in human cancer cell lines where MYXV is permissive and can complete the full virus replication cycle and make progeny virions (for example, A549 lung epithelial carcinoma and 786-0 renal carcinoma cells), DHX9 knockdown further increased progeny virus titer when infected at a low multiplicity of infection (MOI) (i.e., facilitated spread of progeny virus). Using live microscopy, we now show that DHX9 knockdown in the 786-0 cell line resulted in enhanced cell-cell virus spread and formation of larger foci when infected at an MOI of 0.01 (Fig. 3A and Movies S1 and S2). We further extended these observations in nonpermissive human PANC-1 pancreatic cancer cells, where MYXV infection alone makes few if any progeny virions, and tested whether DHX9 knockdown enhanced MYXV progeny virion formation. We also analyzed whether the DHX9-mediated block against MYXV in these cells is at the viral transcriptional or translational level. Four individual DHX9-targeted small interfering RNAs (siRNAs) were independently transfected in PANC-1 cells and then infected with different reporter MYXV constructs to monitor and measure the progression of the virus replication cycle (Fig. 3B). DHX9 protein knockdown was confirmed by Western blot analysis (Fig. 3E). Among the DHX9 siRNAs, two significantly enhanced MYXV replication (more than 2 logs) at both high (5.0) (Fig. 2D) and low (0.5) MOIs (Fig. 3C), suggesting that DHX9 can function as a major cellular restriction factor for MYXV replication in human cells, if the DHX9 antiviral pathway is operationally intact. We further analyzed the impact of the DHX9 inhibitory role on viral protein synthesis. A549 cells were transfected with a nontargeting siRNA as the control or DHX9 siRNA no. 2 and infected with vMyx-FLuc (Fig. 4A) or vMyx-M029KO-FLuc (Fig. 4B), viruses that express FLuc under poxvirus synthetic early/late promoter. vMyx-M029KO-FLuc virus infection is used as the control for showing restriction of late protein synthesis and progeny virus formation. In addition, cells were also pretreated with AraC to specifically block continued expression of FLuc under the viral late promoter. The results indicate that knockdown of DHX9 enhanced FLuc expression from vMyx-FLuc expression at both early and late time points; however, at late time points, FLuc expression increased almost 10-fold compared to untreated virus infection alone or control siRNA transfection. This enhanced FLuc expression at late time points was not observed when cells were infected with vMyx-M029KO-FLuc or pretreated with AraC (Fig. 4B). These results demonstrate that DHX9 antiviral granules, when formed, mostly inhibit the synthesis of late viral proteins. A similar increase in late protein synthesis was observed in nonpermissive human PANC-1 cells after DHX9 knockdown (not shown).

**FIG 5:**
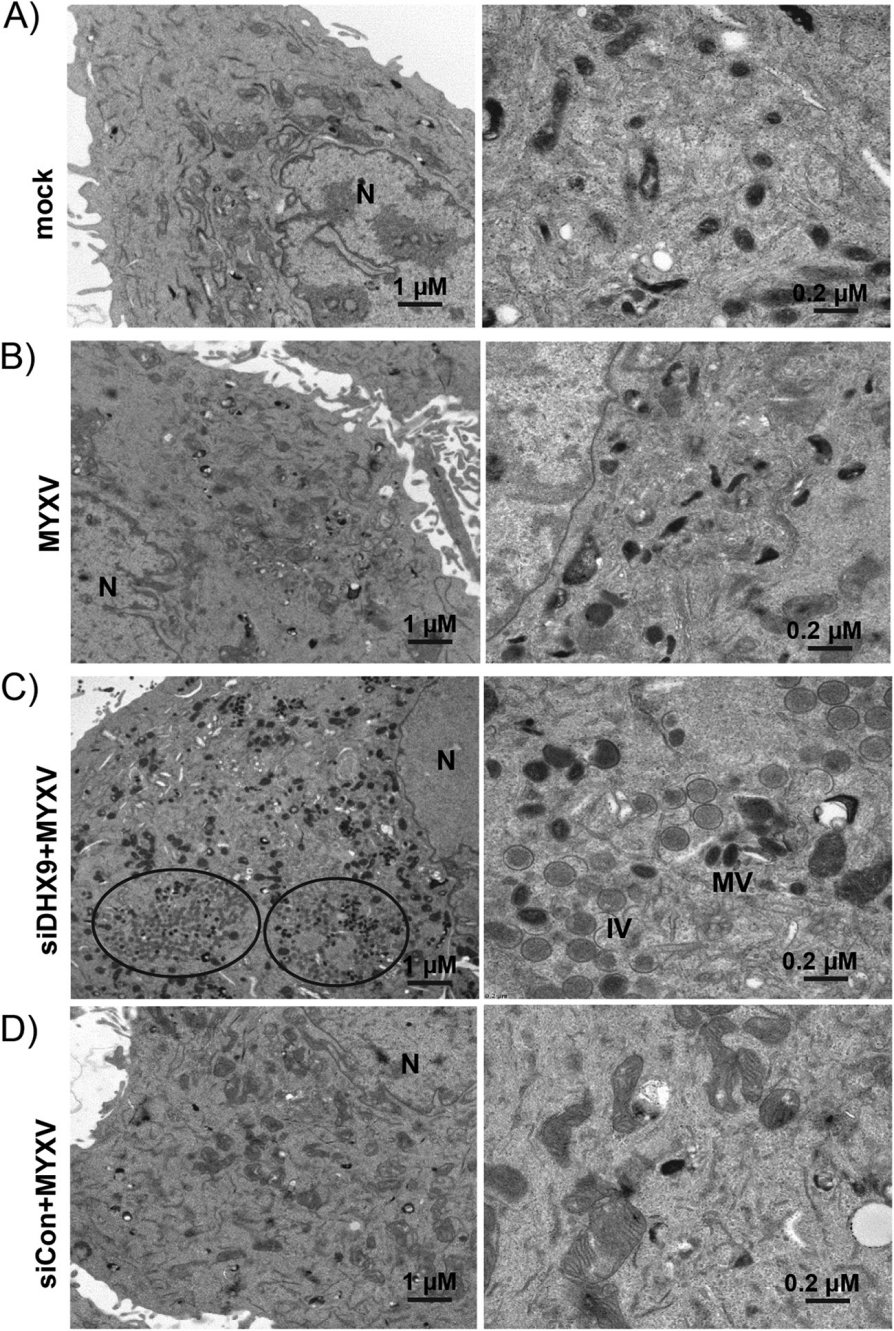
Electron micrograph of MYXV-infected PANC-1 cells. (A to D) Human PANC-1 cells were mock-infected (A) or infected with MYXV alone (B) or after transfection of DHX9 siRNA (C) or a nontargeting control siRNA (D) for 48 h. After 24 hpi, the cells were processed for electron microscopy as described in Materials and Methods. N, nucleus; IV, immature virions; MV, mature virions.

Since DHX9 antiviral granules reduced MYXV late protein synthesis in human cancer cells, we also checked whether transcription of MYXV-carrying genes is inhibited. We performed reverse transcriptase quantitative PCR (RT-qPCR) for selected early (M010 and M013), intermediate (M053), and late (M026) genes in the RNA isolated from control or DHX9 knockdown cells. Knockdown of DHX9 significantly increased both early and late gene transcription; however, the increase in late transcripts was several orders of magnitude higher than that of early gene transcripts (Fig. 4C). This increase in viral late transcripts also correlated with the increase in viral late protein synthesis. We further confirmed that knockdown of DHX9 can enhance progeny virus formation in the restricted PANC-1 cells by using scanning electron microscopy. We observed that MYXV infection after knockdown of DHX9 with DHX9 siRNA, but not control siRNA or MYXV infection alone, allowed formation of distinct viral factories and also resulted in the formation of cytoplasmic mature virions (Fig. 5).

### DHX9 antiviral granules reduce nascent protein synthesis in the MYXV-infected human cancer cells.

**FIG 6:**
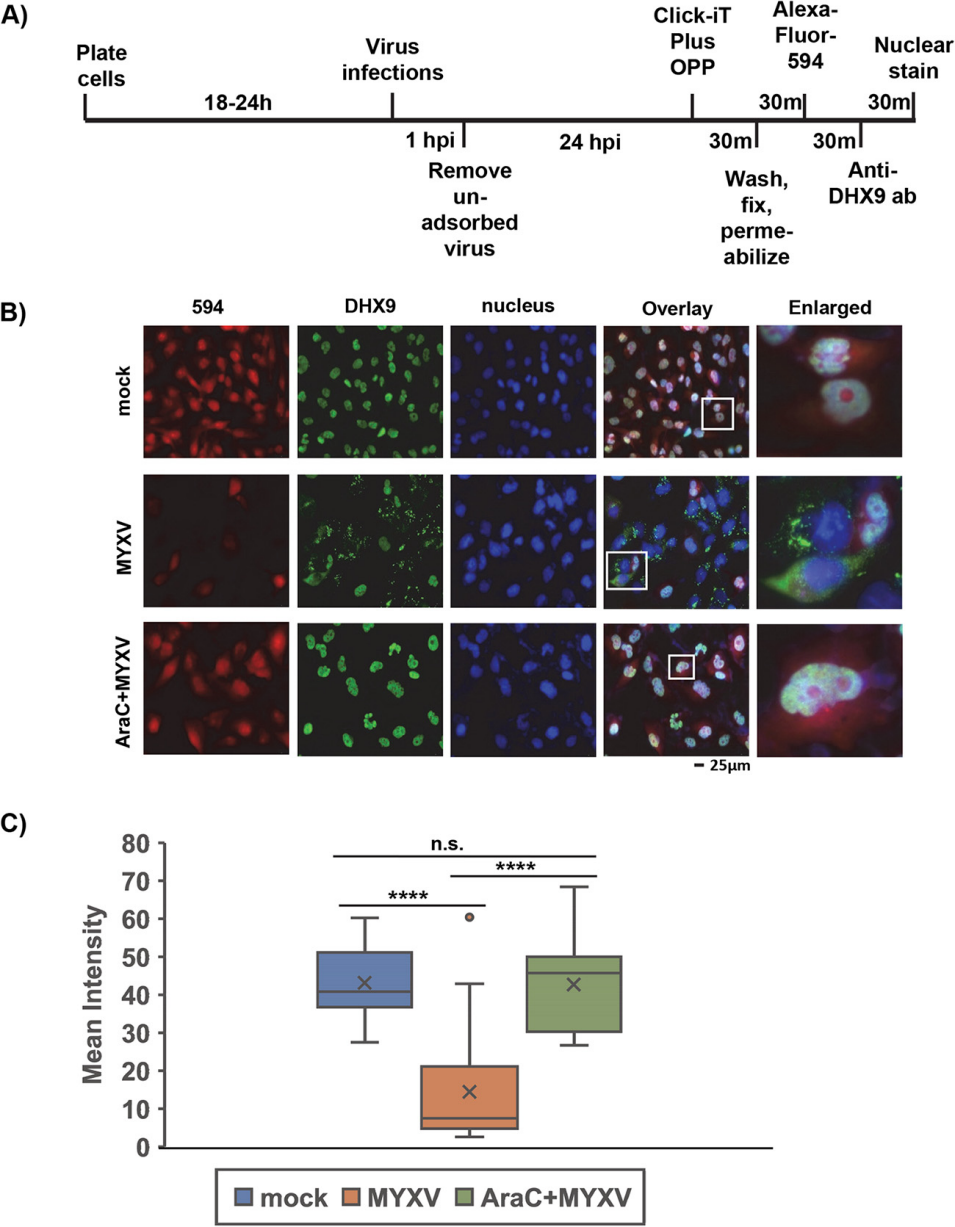
DHX9 antiviral granules reduce nascent protein synthesis in the infected cells. (A) Schematic representation of the newly synthesized protein-labeling protocol using a Click-iT OPP kit. (B) 786-0 cells were either mock-infected or infected with MYXV in the presence or absence of AraC, and newly synthesized proteins were labeled with OPP and processed for staining. The cells were also stained with anti-DHX9 antibody. The images were taken using a fluorescence microscope. (C) Quantification of the mean fluorescence intensity of cells. A minimum of 40 cells were used for analysis from multiple fluorescence images taken in panel B; ****, *P* < 0.0001; ns, not significant.

**FIG 7:**
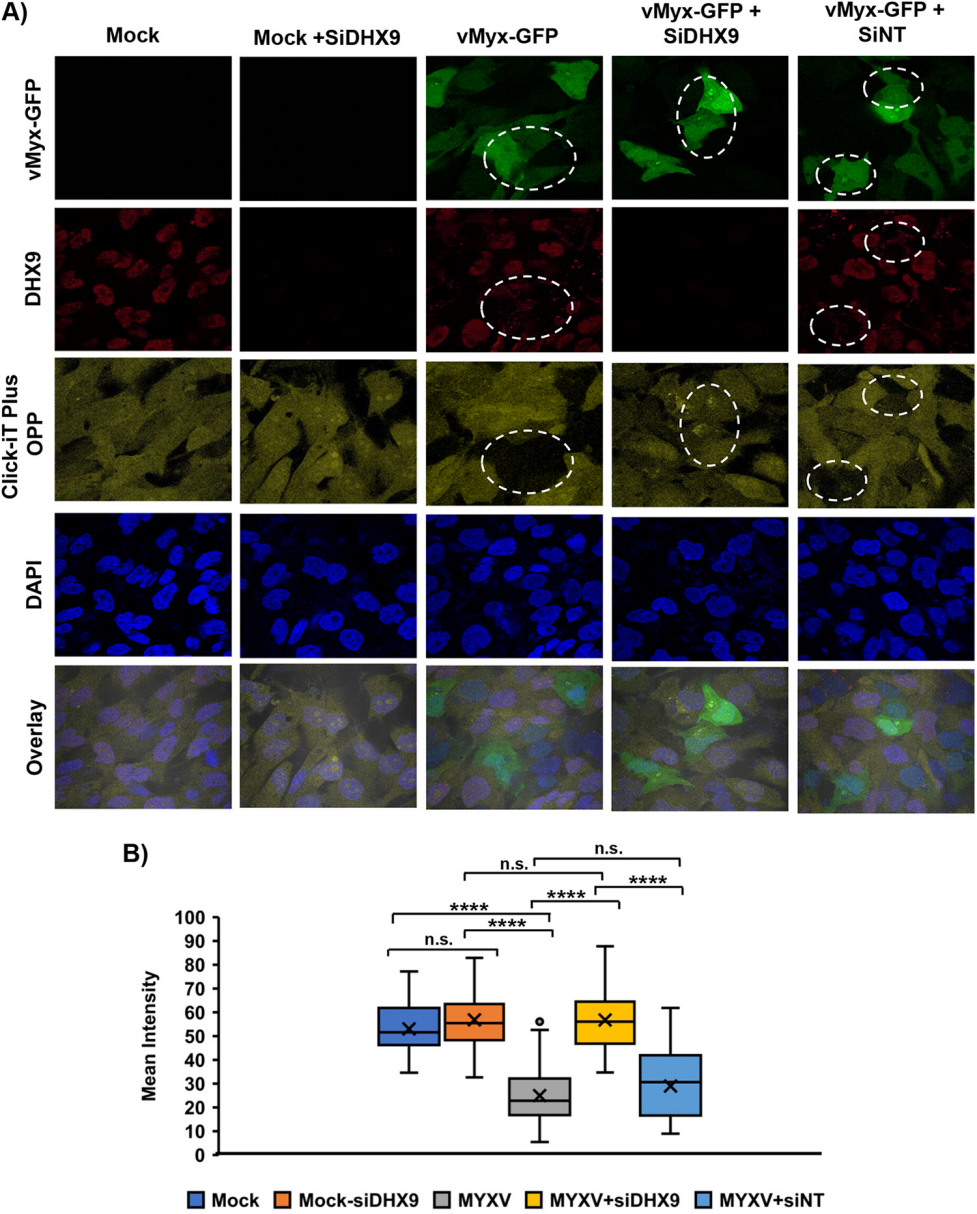
DHX9 knockdown restores nascent protein synthesis in the infected cells. (A) 786-0 cells were transiently transfected with DHX9 siRNA (siDHX9) or nontargeting control siRNA (siNT). After 48 h, the cells were either mock-infected or infected with vMyx-GFP. After 24 hpi, newly synthesized proteins were labeled with Click-iT OPP and processed for staining. The cells were also stained with anti-DHX9 antibody. The images were taken using a confocal microscope. MYXV-infected cells are shown in the circles. (B) Quantification of the mean fluorescence intensity of cells. A minimum of 40 cells were used for analysis from multiple fluorescence images; ****, *P* < 0.0001; ns, not significant.

Having observed that DHX9 reduced the level of viral protein in the infected cells, we measured changes in nascent protein synthesis in A549 cells infected with wild-type MYXV (WT-MYXV) and compared them with cells treated with AraC before infection to block viral late protein synthesis and replication. We found that WT-MYXV infection caused a significant decrease in nascent protein production, as observed and measured using Click-iT Plus OPP staining (Fig. 6A to C). Those cells where protein synthesis decreased also formed the DHX9 antiviral granules in the cytoplasm (Fig. 6B, enlarged images). However, the cells treated with AraC showed a very minimum or no decrease in nascent protein production and no changes in the localization of DHX9 (Fig. 6C). We then tested whether knockdown of DHX9 will restore the nascent protein synthesis in the infected cells. We performed Click-iT OPP staining in the MYXV-infected cells after DHX9 knockdown. The results clearly demonstrated that unlike only MYXV infection or cells transfected with control siRNA, knockdown of DHX9 completely restored the nascent protein synthesis in the MYXV-infected cells (Fig. 7A and B). These results confirm that DHX9 antiviral granules are involved in the reduction of protein synthesis.

**FIG 8:**
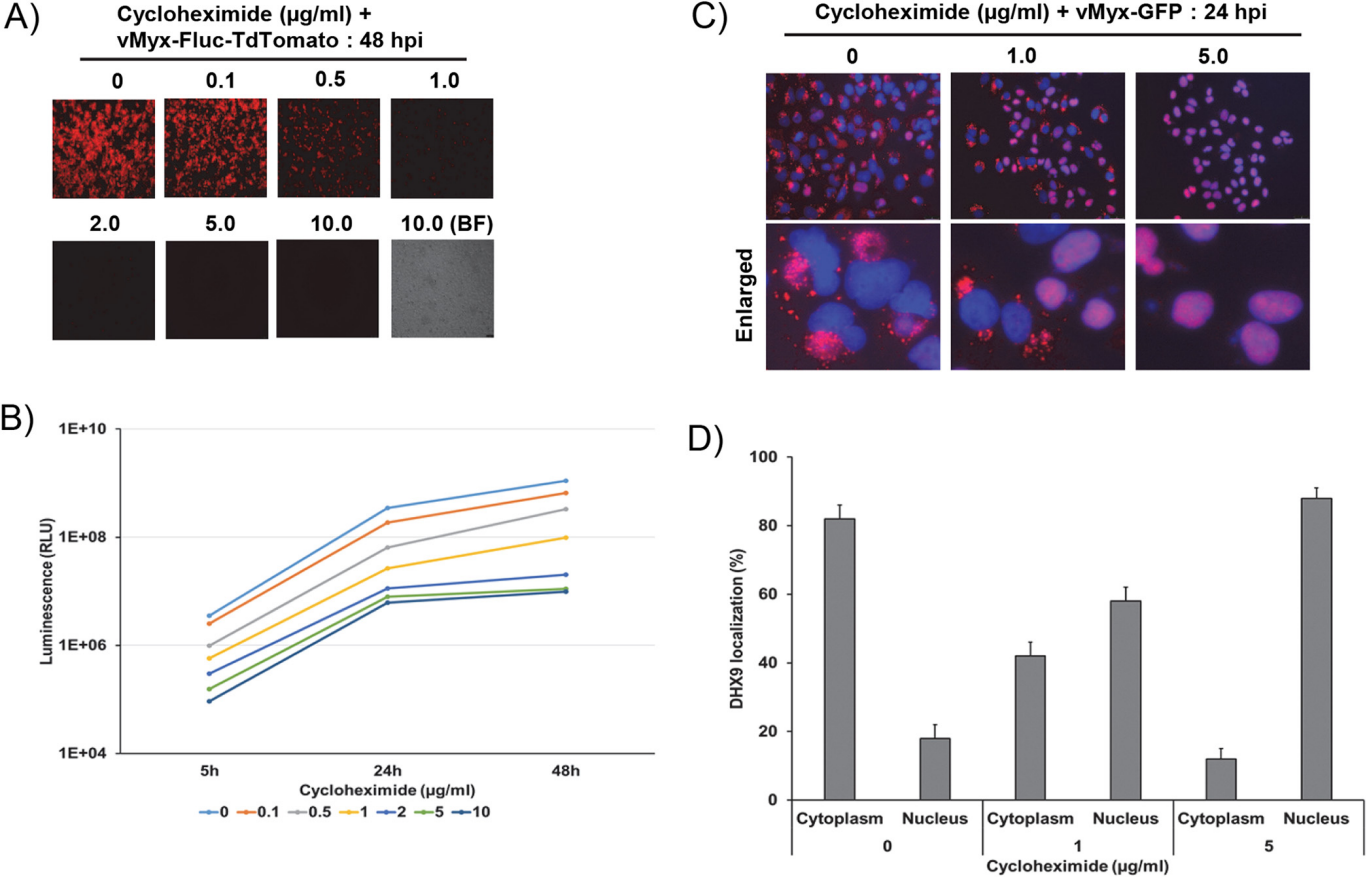
Inhibition of viral protein synthesis reduces the formation of DHX9 antiviral granules. (A) A549 cells were treated with different concentrations of cycloheximide for 1 h and infected with vMyx-Fluc-TdTr (MOI = 1), and fluorescence images were taken at 48 hpi. (B) Cells were also collected at the indicated time points and processed for the luciferase assay. The assays were done in triplicate. A549 cells were seeded on glass-bottom 35-mm petri dishes. The next day, cells were treated with the indicated concentration of cycloheximide for 1 h and infected with vMyx-GFP (MOI = 1.0). After 24 hpi, the cells were fixed and stained with antibodies against DHX9. (C) Nuclei were stained with DAPI and imaged by fluorescence microscope. (D) The number of cells showing strong nuclear or cytoplasmic staining of DHX9 after 24 hpi. A minimum of 100 cells were used for analysis from fluorescence images taken in panel C.

Next, we checked whether a reduction of viral protein synthesis in the cytoplasm will reduce the formation of DHX9-specific antiviral granules and altered localization of DHX9 in the infected cells. For this, we treated cells with cycloheximide, a known inhibitor of cellular and viral protein synthesis. We determined the concentration of cycloheximide that significantly reduced viral early and late protein synthesis without causing immediate cytotoxicity to the cells. A549 cells were treated with increasing concentrations of cycloheximide and then infected with reporter virus vMyx-Fluc-TdTomato (MYXV expressing FLuc under a synE/L promoter and TdTomato under a late p11 promoter). At a concentration of 5 μg/ml or higher, cycloheximide significantly reduced the late protein synthesis as observed from expression of TdTomato under a fluorescence microscope (Fig. 8A). This was also confirmed by measuring the level of FLuc, as there was minimal change in reduction between 5 and 10 μg/ml after 24 hpi (Fig. 8B). Next, we checked whether the cycloheximide treatment-mediated reduction of viral protein synthesis affected the DHX9 localization and formation of antiviral granules containing DHX9. With increasing concentration of cycloheximide, DHX9 was localized in the nucleus in most of the cells (>80%), suggesting that late viral protein synthesis is required for DHX9 altered localization and formation of antiviral granules in the cytoplasm of MYXV-infected cells (Fig. 8C and D).

### DHX9 is not a component of the cellular stress granules.

**FIG 9:**
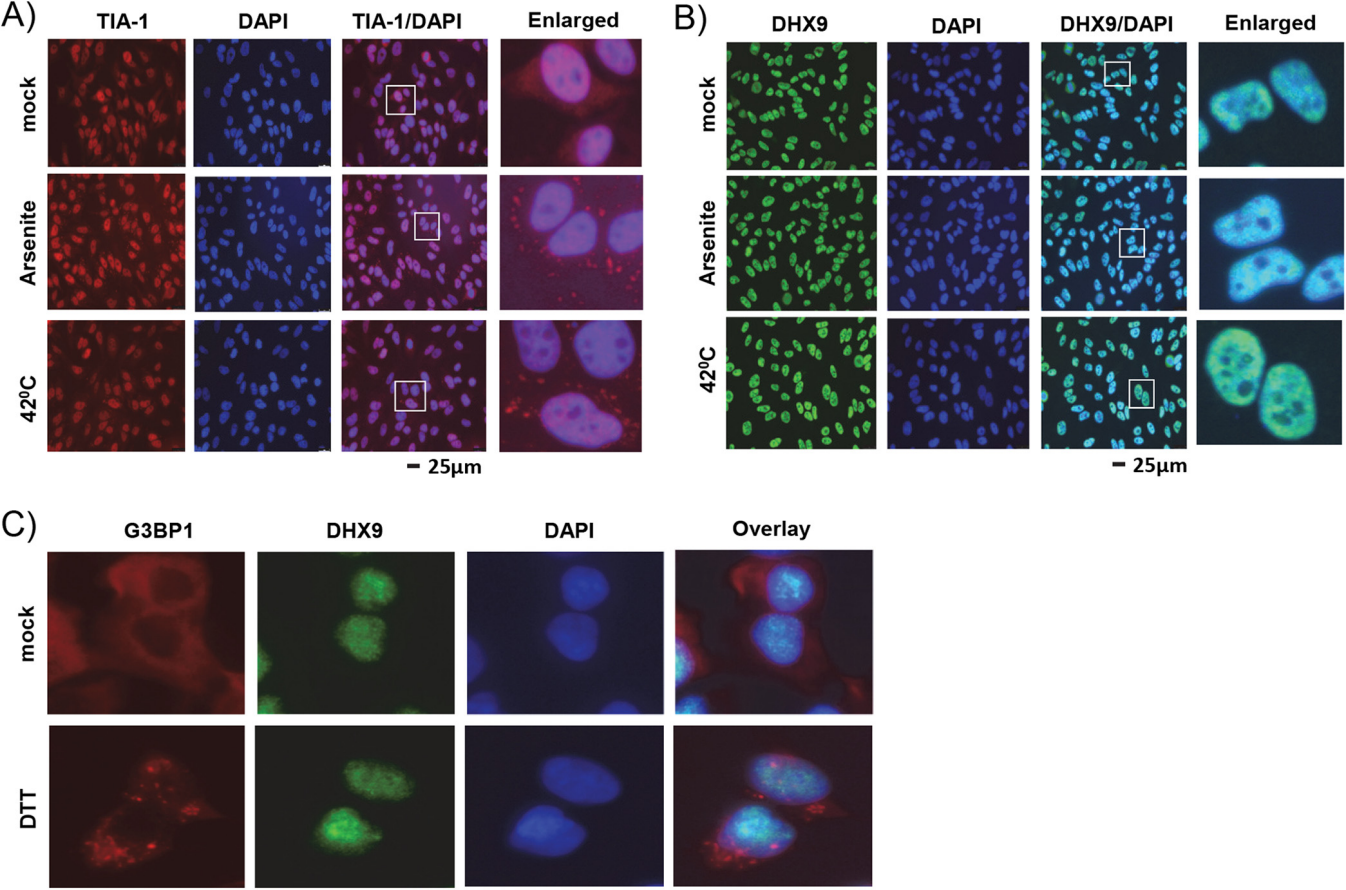
DHX9 is not associated with cellular stress granules. A549 cells were seeded on glass-bottom 35-mm petri dishes and left overnight to adhere. The next day, cells were left untreated or treated for the formation of stress granules. (A and B) Cells were treated with arsenite or incubated at 42°C for 30 min, fixed, and stained with antibodies against TIA-1 (A) or DHX9 (B). Nuclei were stained with DAPI. (C) A549 cells were left untreated or treated with DTT for 2 h, fixed, and stained with antibodies against G3BP1 and DHX9. Nuclei were stained with DAPI. Images were taken using a fluorescence microscope.

DHX9 has not previously been reported to be associated with any cytoplasmic stress granules. Therefore, we first checked whether DHX9 is a component of canonical stress granules formed by other nonviral stress-inducing conditions, such as arsenite or heat shock (37). After treatment of A549 cells with arsenite or incubation at 42°C, the localization and formation of stress granules were followed using immunofluorescence staining of known stress granule components. Both arsenite and heat shock treatment allowed formation of cytoplasmic stress granules positive for TIA-1, a known component of these stress granules (Fig. 9A). In the mock-treated control A549 cells, TIA1 was present mainly in the nucleus; upon treatment with stress inducers, TIA1 was detected in the stress granules (Fig. 9A). However, these nonviral treatments did not change the nuclear localization of DHX9 or association of DHX9 with the distinct DHX9-containing cytoplasmic stress granules (Fig. 9B). We further confirmed this by treatment of cells with another known stress inducer, dithiothreitol (DTT). After treatment of cells with DTT, they were costained with antibodies against DHX9 and G3BP1, a known component of cellular stress granules. Again, in these DTT-treated cells, only G3BP1 was detected in the stress granules, but not DHX9. These results clearly demonstrate that DHX9 is not associated with the classic stress granules formed by treatment with cellular stress inducers after accumulation of stalled protein translation, but is instead associated with novel antiviral granules induced by poxvirus replication in the cytoplasm reported in this paper.

### Stress granule components are associated with DHX9 antiviral granules.

**FIG 10:**
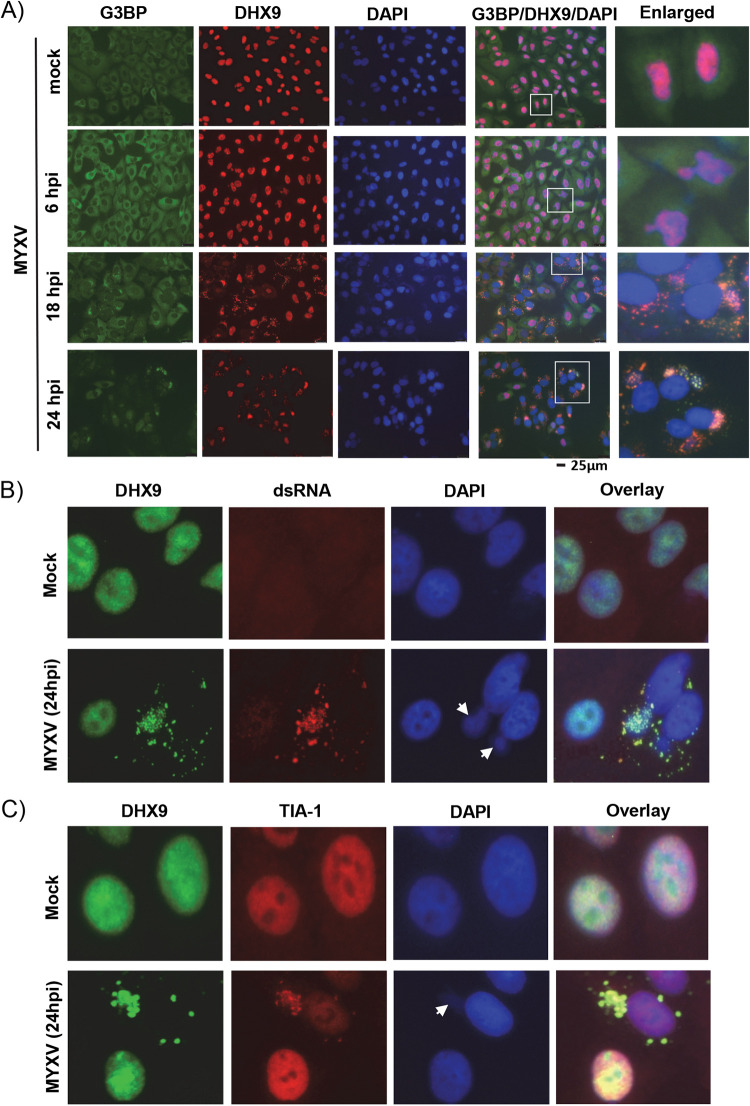
DHX9 antiviral granules are associated with stress granule components. A549 cells were seeded on glass-bottom 35-mm petri dishes and left overnight to adhere. The next day, cells were mock-infected or infected with WT-MYXV (MOI = 1.0). (A to C) At the indicated time points, cells were fixed and stained with antibodies against (A) DHX9 (red) and G3BP1 (green), (B) DHX9 (green) and dsRNA and (C) DHX9 (green) and TIA-1 (red). Nuclei were stained with DAPI. Viral factories stained with DAPI are indicated with arrows.

It was reported that cytoplasmic stress granules can form during poxvirus infection, which compete with the viral factories (38). In order to understand whether the DHX9 antiviral granules are indeed associated with the proteins of the cellular stress granules, and whether they might be sequestering the viral protein synthesis machinery, we have used immunofluorescence microscopy to localize known stress granule components such as G3BP1 and DHX9 in uninfected and MYXV-infected cells. In uninfected A549 cells, G3BP1 is mostly localized in the cytoplasm (37). When human cells were infected with MYXV, even after 6 hpi when viral factories first formed and DHX9 was relocalized in the viral factories, G3BP1 was still detected in the cytoplasm as in mock (noninfected control) cells. However, at late time points (18 and 24 hpi), G3BP1 is seen to become associated with viral factories along with DHX9 in the infected cells (Fig. 10A, bottom two panels). Again, treatment with AraC completely blocked the formation and colocalization of DHX9 and G3BP within the same granules (not shown). This suggests that the formation of these granular structures is late viral protein dependent. We further confirmed the presence of other stress granule components such as TIA-1 in the DHX9 antiviral granules (Fig. 10C). Apart from components of the cellular stress granules, we also confirmed the colocalization of dsRNA (which were detected upon viral infection) in the DHX9 antiviral granules (Fig. 10B). In the uninfected cells, this antibody did not give any signal; however, dsRNA was detected only in the infected cells, and they were colocalized with DHX9 antiviral granules.

### MYXV M029 host range protein also localizes in the DHX9 antiviral granules.

**FIG 11:**
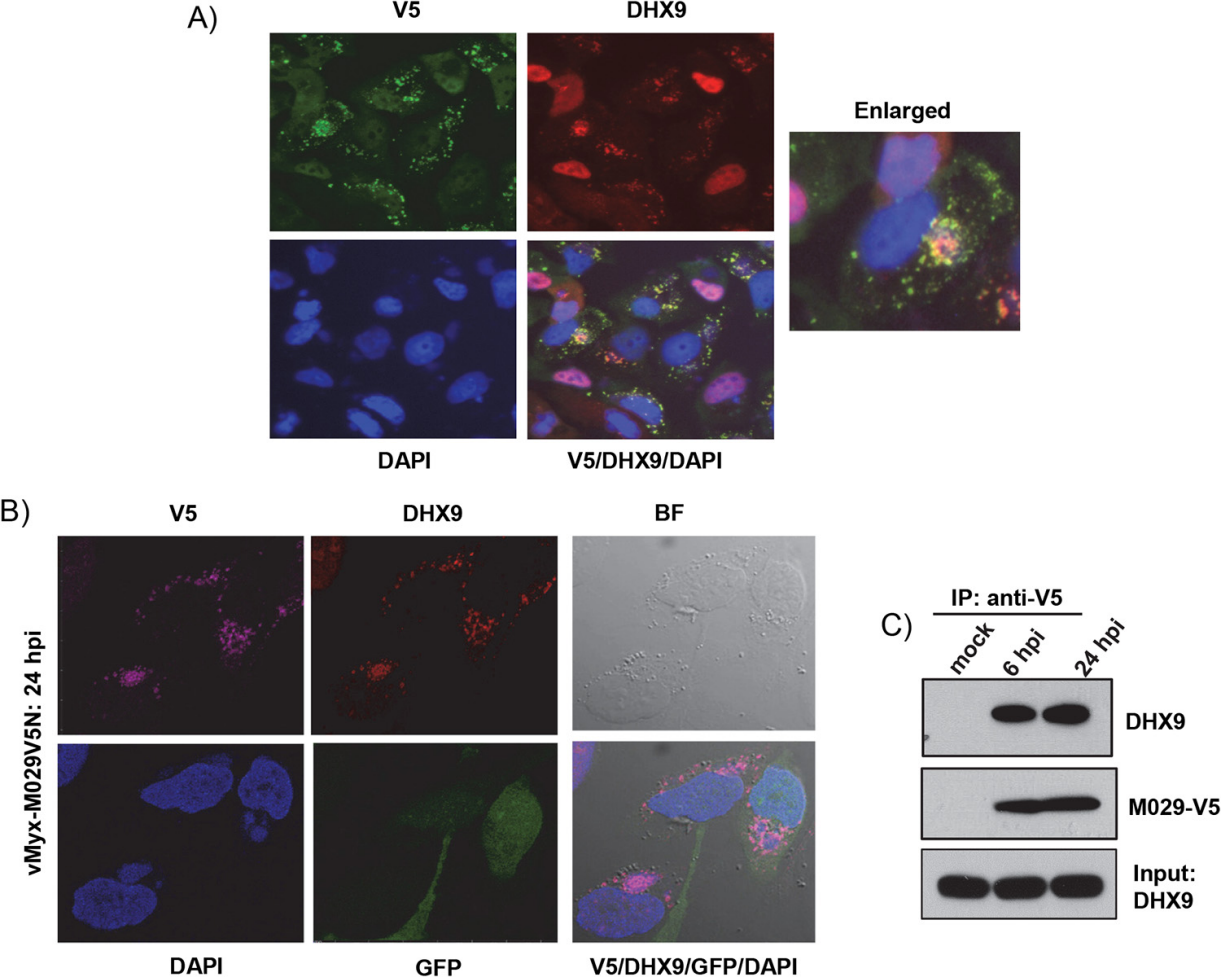
M029 and DHX9 are colocalized in the antiviral granules. (A) A549 cells were seeded on glass-bottom 35-mm petri dishes and left overnight to adhere. The next day, cells were infected with WT-MYXV for 1 h and transfected with plasmid having V5-tagged M029 under the synE/L promoter. After 24 h, cells were fixed and stained with antibodies against DHX9 (red) and V5 (green). Nuclei were stained with DAPI. (B) A549 cells seeded on glass-bottom 35-mm petri dishes were infected with vMyx-M029V5N. After 24 h, cells were fixed and stained with antibodies against DHX9 (red) and V5 (maroon) and imaged using a confocal microscope. (C) A549 cells were infected with vMyx-M029V5N, and cell lysates were collected at the indicated time points. Co-IP was performed using anti-V5 antibody. After Western blot analysis, the membrane was probed with different antibodies.

We reported previously that MYXV host range protein M029, a dsRNA binding protein, interacts physically with DHX9 protein (9). We now show that in the absence of M029, DHX9 mostly remains in the nucleus and does not relocalize to form the cytoplasmic antiviral granules (Fig. 1D). We next tested whether M029 is also localized to DHX9 antiviral granules. We used two approaches to study localization of M029 and DHX9 during MYXV infection. In the first approach, we used transient transfection using a plasmid expressing N terminus V5-tagged M029 under poxvirus SynE/L promoter. In A549 cells after infection with WT-MYXV and transfection of this plasmid, expression of M029 was detected with anti-V5 antibody. M029 can be clearly observed in the cytoplasmic granular structures both in the viral factories and cytoplasm after 24 hpi, similar to the distribution profile of DHX9 (Fig. 11A). When the same cells were stained for DHX9, the same granules were also positive for DHX9, suggesting that M029 and DHX9 are colocalized in the same antiviral granules. This colocalization of DHX9 and M029 was also confirmed after infection of cells with vMyx-M029V5N, a MYXV expressing N terminus V5-tagged M029 under the native viral promoter (9). The infected cells are GFP positive, and the viral factories are visualized with DAPI staining. In these cells, confocal images again clearly demonstrate that M029 and DHX9 are colocalized in both the viral factories and the DHX9-specific antiviral granules formed in the cytoplasm (Fig. 11B). The interaction between M029 and DHX9 was also confirmed by coimmunoprecipitation after infection of cells with vMyx-M029V5N (Fig. 11C).

## DISCUSSION

An in-depth understanding of virus-cancer cell interactions is crucial for the development of therapeutically effective oncolytic virotherapy. Our recent findings that multiple host RNA helicases play a major role in the tropism of oncolytic MYXV in a subset of human cancer cells highlight the importance of uncovering how this family of essential host regulatory proteins varies in normal versus cancerous cells and how their antiviral activities can influence MYXV and other oncolytic viruses (11). In the present study, our focus was to understand how RHA/DHX9, a key member of cellular RNA helicases, restricts MYXV infection and replication in a subset of MYXV-resistant or semipermissive human cancer cell types. Based on MYXV infection and replication in human cancer cells, they can be classified as permissive (supporting high levels of progeny virions, comparable to fully permissive rabbit cells), semipermissive (capable of producing lower levels of progeny virions, generally less than 10 infectious units per cell), and nonpermissive (essentially no progeny virion synthesis) (7, 39, 40). We recently reported that knockdown of certain members of RNA helicases, including DHX9, in permissive, semipermissive, or nonpermissive human cancer cell lines enhanced MYXV replication and progeny virus formation in many members of each cell class, suggesting that cellular helicases such as DHX9 can act as viral restriction factors for MYXV in some, but not all, human cancer cells (11).

In order to understand the mechanisms how RNA helicases such as DHX9 restrict MYXV replication only in selective human cancer cells, we first studied the localization of DHX9 in virus-infected cells. Importantly, we observed that the nuclear localization of DHX9 (where DHX9 mostly remains in the uninfected control cells) was totally reconfigured during MYXV infection. DHX9 was detected associated with viral factories after cytoplasmic viral factories form, then accumulated in these viral factories, and subsequently formed distinct DHX9-containing antiviral granules in the cytoplasm during the viral late replication cycle. We further confirmed that this formation of DHX9 antiviral granules can be completely blocked using AraC, an inhibitor of poxviral DNA replication and late protein synthesis. Additionally, infection with vMyx-M029KO virus, which is highly defective in late protein synthesis in human cells, also significantly reduced DHX9 antiviral granules. DHX9 protein possesses well-characterized nuclear export and import signals, which allow DHX9 to be cycled between the nucleus and cytoplasm as needed for performing diverse cellular functions (15). For example, nuclear import of DHX9 is mediated by a Ran-dependent classical importin-alpha/beta-dependent pathway (41). Additionally, DHX9-encoded dsRNA binding domains I and II (dsRBD I and II) are also important for cytoplasmic localization (42). Different subcellular localizations of DHX9 have been reported for RNA viruses, which can target and exploit DHX9 to permit optimal replication in either the nucleus or cytoplasm. For example, during BVDV virus replication, DHX9 is exported to the cytoplasm of the infected cells by viral N-terminal protease N(pro) (43). PRRSV N protein also interacts with DHX9 and redistributes the DHX9 in the cytoplasm (44). These interactions of RNA viruses with DHX9 and virus-specified recruitment mainly allow optimal viral RNA synthesis and virus production. In contrast to RNA viruses, very little is known about the function of DHX9 during DNA virus replication. Our results clearly demonstrate that, unlike RNA viruses, DHX9 formation of the cytoplasmic granular structures in human cells can significantly restrict MYXV replication in at least a subset of human cancer cells. This is particularly significant because in human cancer cells that are nonpermissive for MYXV, for example, PANC-1 human pancreatic cancer cells, DHX9 knockdown alone increased MYXV replication by more than 2 orders of magnitude. This suggests that selective targeting of DHX9 can potentially enhance oncolytic virotherapy of MYXV, and possibly other poxviruses, in a subset of cells that are not naturally permissive for the virus.

The DHX9 antiviral granules that form at the late stage of MYXV replication negatively impact viral gene transcription and translation, since knockdown of DHX9 significantly increased both and amplified progeny virus synthesis. This suggests that DHX9 antiviral granules, by sequestering selective viral transcriptional and translational machinery elements from the cytoplasmic viral factories, causes reduced progeny virus formation. The observation that DHX9 antiviral granules reduced viral protein synthesis was further confirmed by monitoring nascent protein synthesis in the infected cells using immunofluorescence labeling of nascent protein synthesis. The results confirmed that in the cells, where DHX9 formed antiviral granules, the nascent protein synthesis was significantly reduced. These results clearly demonstrated that DHX9 antiviral granules restrict MYXV replication by inhibition of protein synthesis.

To confirm that viral late protein synthesis triggers the formation of DHX9 antiviral granules, we used protein synthesis inhibitor cycloheximide to study the effect of full translational blockade on DHX9 localization. As expected, treatment with increasing doses of cycloheximide reduced viral late protein synthesis in the MYXV-infected cells and also, in concert, progressively reduced DHX9 antiviral granule formation. Apart from virus infection, since there is no report that DHX9 is associated with classical cytoplasmic stress granules that are formed due to stalled protein synthesis, we first tested whether in these cells, the stress granules formed by known stress inducers such as arsenite, heat shock, or dithiothreitol also contain DHX9. Our results clearly demonstrate that, although stress granule-associated protein TIA1 or G3BP1 (45) can be detected in these canonical stress granules, DHX9 is not associated with canonical cellular stress granules. However, we cannot totally rule out the possibility that DHX9 is associated with cellular stress granules based on the dynamic function of DHX9 and the presence in both the nuclear and cytosolic fractions of cells. Indeed, the presence of DHX9 was reported in a study where >300 stress granule-associated proteins were identified using mass spectrometric analysis (46).

Virus infection also results in the formation of classic TIA1/G3BP1-positive stress granules due to viral protein synthesis and replication (47). We checked whether G3BP1, a cytoplasmic protein also associated with stress granules, is present in DHX9 antiviral granules. Immunostaining data demonstrated that G3BP1 formed granular structures during the late replication cycle of MYXV and that when colocalized with DHX9, both G3BP1 and DHX9 were present in the same antiviral granules. Apart from G3BP1, the DHX9 antiviral granules were also colocalized with TIA-1. Unlike G3BP1, TIA-1 is mainly localized in the nucleus; however, in the infected cells, TIA-1 become associated with the DHX9 antiviral granules. Apart from the known stress-associated cellular proteins, DHX9 antiviral granules were also associated with dsRNA, which is produced during virus replication. We further confirmed that the MYXV dsRNA binding protein M029 is also present in the DHX9 antiviral granules. Thus, the DHX9 antiviral granules function by hijacking viral components and by inhibition of *de novo* protein synthesis.

In conclusion, our study demonstrates a novel role for DHX9 in restricting oncolytic MYXV replication in human cancer cells. DHX9 antiviral granules reduced viral protein synthesis in all the human cancer cell types we have tested, albeit at variable levels. However, in the absence of DHX9, we observed significantly enhanced rescue in viral protein synthesis, replication, and progeny virus production in nonpermissive human cancer cell lines. In these nonpermissive cancer cell lines, apart from DHX9 antiviral granules, other signaling pathways or cellular factors, for example, the level of activated/phosphorylated AKT, also contribute to the restrictive replication of MYXV (7, 48). It would be interesting to test in the future whether these cellular factors or pathways are linked with the formation of DHX9 antiviral granules or operate independently to contribute to the nonpermissiveness in selected cancer cell types. Another possibility is that DHX9-mediated antiviral pathways are fully operational in certain cancer cell types and defective in many, if not most, human cancer cells, thus allowing greater levels of MYXV replication than in their normal somatic cell counterparts. As a corollary, we propose that the M029 host range protein of MYXV binds and inhibits DHX9 effectively in rabbit cells but is an imperfect inhibitor of the same pathway in human cells. Additionally, the subset of MYXV-restricted human cancer cells where the DHX9 pathway is fully active can be converted from a restrictive to a more MYXV-permissive phenotype by selectively inhibiting DHX9 or by blocking the signaling pathway. Thus, these results have direct implications for successful oncolytic virotherapy against even the subset of normally MYXV-resistant cancers.

## MATERIALS AND METHODS

### Cell culture.

Rabbit cell line RK13 (ATCC CCL-37), RK13 expressing VACV E3 protein (RK13-E3) (9), and human cell lines HeLa (ATCC CCL-2), A549 (ATCC CCL-185), PANC-1 (ATCC CRL-1469), and 786-0 (ATCC CRL1932) all were cultured in Dulbecco minimum essential medium (DMEM; Invitrogen) supplemented with 10% fetal bovine serum (Sigma and others), 2 mM glutamine (Invitrogen), and 100 μg/ml of penicillin-streptomycin (pen/strep; Invitrogen). All cultures were maintained at 37°C in a humidified 5% incubator.

### Viruses.

WT-MYXV (MYXV Lausanne), vMyx-GFP (WT-MYXV that expresses GFP under poxvirus synthetic early/late promoter [sE/L]), vMyx-GFP-Tdtomato (WT-MYXV that expresses GFP under sE/L promoter and Tdtomato under a poxvirus p11 late promoter), vMyx-FLuc (WT-MYXV that expresses firefly luciferase under poxvirus sE/L promoter and Tdtomato under poxvirus p11 late promoter), vMyx-Venus/M093 (WT-MYXV that expresses Venus-tagged M093 protein as a virion component), and vMyx-M029KO-Fluc (M029-KO virus that expresses firefly luciferase under poxvirus sE/L promoter and Tdtomato under poxvirus p11 late promoter) virus constructs, as described before, were used (11, 49, 50). The M029-minus viruses were grown and amplified in RK13-E3 cells. All other myxoma viruses were grown in RK13 cells. The virus stocks used were prepared using sucrose gradient purification as described before (51).

### Reagents and antibodies.

Mouse monoclonal antibody for DHX9, G3BP1, and goat polyclonal antibody for TIA1 were purchased from Santa Cruz Biotechnology. Rabbit polyclonal antibody for DHX9, mouse antibody for V5, and β-actin were purchased from Thermo Fisher Scientific. Cycloheximide and AraC were purchased from Sigma. Mouse monoclonal antibody for dsRNA SCICONS J2 was from English and Scientific Consulting Kft., Hungary (SCICONS). Horseradish peroxidase (HRP)-conjugated goat anti-rabbit and anti-mouse IgG antibodies were purchased from Jackson ImmunoResearch Laboratories. All the secondary antibodies conjugated to Alexa Fluor 488, 594, 568, and 647 were purchased from Thermo Fisher Scientific.

### RNA isolation and real-time PCR (qPCR).

For isolation of RNA, 1 × 10^6^ cells were plated in each well of six-well dishes. The following day, cells were infected with the viruses at an MOI of 1. Cells were harvested at 6 h and 24 h after infection. Total RNA isolation, cDNA preparation, and real-time PCR were performed based on the protocol described before (5). The PCRs were run on a ViiA 7 real-time PCR system under the following conditions: 95°C for 10 min, followed by 40 cycles of 95°C for 15 s and 60°C for 1 min. The primers used for real-time PCR analysis were human GAPDH (forward [F], GTGGACCTGACCTGCCGTCT; reverse [R], GGAGGAGTGGGTGTCGCTGT), M13 (F, GGAGGACCTATACATCGAAC; R, CGCCAATAGGGAATCCACG), M053 (F, GGCGGCTCCGTAAACATATGG; R, TCGGGTTGTATTTCTATTACGG), M026 (F, CGCACATACACCATTTTACATC; R, GGCTTTCGGTCACACGTAG), and M10 (F, TATGTAACGACGACTATAAAAAC; R, CTGACATCGGCTTCCCACG). Amplification of genes was normalized to GAPDH amplification from the same sample, and the fold induction of genes after viral infection was calculated relative to that at 24 hpi and 6 hpi.

### siRNA transfection.

Four independent siRNAs for DHX9, ON-TARGETplus SMART pool siRNAs for G3BP1, and the nontargeting control (siCon) were purchased from Dharmacon. Cells were seeded with 40 to 50% confluence, left overnight for adherence, and then transfected with siRNA (50 nM) using the Lipofectamine RNAiMAX (Invitrogen) transfection reagent. After 48 h of transfection, the cells were infected with the viruses for 1 h, washed to remove unbound virus, and incubated with complete medium. At the indicated time points, cells were either observed under fluorescence microscope to monitor and record the expression of fluorescence proteins or lysed for luciferase assays or harvested and processed for titration of progeny virions.

### Western blot analysis.

Western blot analysis was performed as described before (9). Briefly, the mock- or virus-infected cells were collected at different time points after infection, washed with phosphate-buffered saline (PBS), and processed with RIPA lysis buffer. Nuclear and cytosolic fractions of cells were prepared using NE-PER nuclear and cytoplasmic extraction reagents (Thermo Scientific). Equal amounts of total proteins were used for Western blot analysis. The membranes were first blocked in TBST buffer (20 mM Tris, 150 mM NaCl, 0.1% Tween 20, pH 7.6) containing 5% nonfat dry milk for 1 h at room temperature and then incubated overnight with appropriate primary antibody at 4°C. The membranes were washed with TBST and incubated with HRP-conjugated secondary antibody in TBST containing 5% nonfat dry milk for 1 h at room temperature. The membranes were again washed with TBST, and the proteins were detected using the chemiluminescence substrate (EMD Millipore) and exposure to X-ray film.

### Coimmunoprecipitation (co-IP) of protein samples.

For co-IP, cells were lysed using RIPA lysis buffer. The cleared cell lysates after centrifugation for 15 min at 12,000 rpm at 4°C were incubated with Pierce protein A/G agarose (Thermo Scientific) for preclearing. The agarose beads were removed by centrifugation for 15 min at 12,000 rpm at 4°C. The supernatants were incubated with the antibody for 1 h at 4°C, followed by incubation with protein A/G agarose overnight at 4°C. The agarose beads were pelleted by centrifugation at 2,000 rpm for 2 min and washed four times with lysis buffer, and samples were then analyzed by Western blotting.

### Immunofluorescence.

Cells (5 × 10^5^ to 1 × 10^6^/dish) were seeded on glass-bottom 35-mm petri dishes overnight. Depending on the experiments, cells were transfected with siRNA or plasmid for 48 h or infected with viruses next day. For virus infection, based on MOI, the desired amounts of viruses were added and cells were incubated for 1 h and replaced with fresh medium. After infection, cells were washed with PBS 3 times, fixed with 2% paraformaldehyde in PBS for 12 min at room temperature, washed again with PBS 3 times, and permeabilized in 0.1% Triton X-100 in PBS for 90 sec at room temperature. Cells were washed with PBS 3 times and then blocked with 3% bovine serum albumin (BSA) in PBS for 30 min at 37°C. Cells were incubated with primary antibody (1:300 dilution) for 30 min at 37°C. Cells were washed with PBS 6 times and incubated with secondary antibody conjugated to different fluorescent dyes. Cells were washed with PBS 6 times and mounted on glass slides with Vectashield (Vectorlabs) containing DAPI (4′,6-diamidino-2-phenylindole) to stain DNA in the nuclei and viral factory. Images were captured on a Leica laser scanning confocal microscope or Leica fluorescence microscope.

### Firefly luciferase (Fluc) assay.

The firefly luciferase assays were performed using a 96-well plate. The cells were seeded with 80 to 100% confluence and allowed to adhere on the surface overnight after seeding. The next day, virus containing complete medium was prepared based on the MOI and number of wells. Media in the wells were aspirated and replaced with the virus-containing media. After 1 h of virus adsorption to the cells, fresh medium was added, replacing the virus-containing media. At the indicated time points, luciferase assay was performed using the luciferase reporter assay kit (Promega, Madison, WI, USA). Briefly, medium was removed, and cells were washed with PBS once and added to the 1× lysis buffer. Lysis was done at room temperature (RT) for 15 to 20 min, substrate was added, and a reading was taken immediately using a microplate reader (Thermo Fisher Scientific).

### Electron microscopy.

Human PANC-1 cells were seeded on ACLAR film (Ted Pella, Inc.) placed in 24-well multiwell dishes. In selected wells, cells were transfected with DHX9 siRNA for 48 as described in the siRNA transfection protocol and then infected with MYXV. In other wells, cells were either mock-infected or treated with leptomycin B (0.01 μM) for 1 h before infection with MYXV with an MOI of 5.0. After 1 h of infection, the virus was removed and washed, and cells were incubated with fresh media. Then, 48 h postinfection, cells were processed for transmission electron microscopy. Cell monolayers were primary-fixed using 2.5% glutaraldehyde in PBS for 3 h on ice, followed by rinsing in PBS and a 45-min incubation on ice with 20 mM glycine in PBS to quench unreacted aldehyde. Samples were then secondary-fixed on ice using 1% osmium tetroxide in PBS for 1.5 h, rinsed well with deionized water, and stained overnight at 4°C with 0.5% aqueous uranyl acetate. Samples were then dehydrated at room temperature using a graded series of acetone solutions and infiltrated with Spurr’s epoxy resin, standard mixture (1). Monolayers were embedded flat in fresh Spurr’s resin under Teflon strips on glass slides and placed in an oven at 60°C for 36 h. Regions of the embedded monolayers were selected using a light microscope and then cut from the slide with a razor and glue-mounted onto a blank resin block so planar-orientation sections could be taken. Sections were cut at 70-nm thickness using a Leica Ultracut-R microtome, and the sections were mounted on Formvar-coated copper slot-grids. Poststaining was done using 2% uranyl acetate in 50% ethanol for 8 min, followed by Sato’s lead citrate (52) for 4 min. Images were generated using a Philips CM12 transmission electron microscope (TEM) at 80 kV and acquired using a Gatan model 791 slow-scan charge-coupled-device (CCD) camera (1,024 × 1,024 pixel resolution) located at ASU life science electron microscopy facility.

### Click-iT Plus OPP protein synthesis assays.

For detection of changes in protein expression, Click-iT Plus OPP (O-propargyl-puromycin) protein synthesis assay kits (molecular probes by Life Technologies) were used. Cells (5 × 10^5^/dish) were seeded on glass-bottom dishes and allowed to adhere by incubation overnight at 37°C. The next day, cells were infected with MYXV for 1 h, and the medium was replaced with fresh medium. After 24 h of incubation, O-propargyl-puromycin (OPP; 20 μM) was added to the cells and incubated for 30 min under the same conditions. Cells were washed with PBS, fixed with 3.7% formaldehyde in PBS, and permeabilized with 0.5% Triton X-100 in PBS as recommended by the supplier. Cells were than incubated for 30 min at room temperature with Click-iT Plus OPP reaction cocktail with Alexa Fluor 594 as described in the manufacturer’s instructions. The cells were washed with PBS and subsequently stained with anti-DHX9 antibody for 30 min at room temperature and HCS NuclearMask blue stain for DNA staining. Fluorescence images were taken using fluorescence and confocal microscopes. The fluorescence images were analyzed using ImageJ (https://imagej.nih.gov/ij/index.html). The images were background subtracted, and the mean nuclear Alexa Fluor 594 fluorescence was quantified for each cell for the Click-iT Plus OPP staining.

### Statistical analysis.

The data show the means and standard deviations of one representative of —two to three independent experiments. Groups were compared using two-tailed Student’s *t* test. *P* values of <0.05 were considered significant.
