# Supplementary material for: RNA Helicase A/DHX9 Forms Unique Cytoplasmic Antiviral Granules That Restrict Oncolytic Myxoma Virus Replication in Human Cancer Cells
Source: J Virol. 2021 Jun 24;95(14):e00151-21. doi: 10.1128/JVI.00151-21 (PMC8223942; doi:10.1128/JVI.00151-21)
Supplement: Supplemental file 6 — Supplemental Movie Legends. Download JVI.00151-21-s0006.pdf, PDF file, 7.50 MB [file jvi.00151-21-s0006.pdf]

Supplemental Material

Supplemental Movie 1. 786-0 cells were infected with vMyx-GFP (MOI = 0.01) for 1h and replaced with fresh media. After 24h live images were captured using Evos microscope.

Supplemental Movie 2. 786-0 cells were transiently transfected with DHX9 siRNA #1. After 48h, the cells were infected with vMyx-GFP (MOI = 0.01) for 1h and replaced with fresh media. After 24h live images were captured using Evos microscope.

Supplemental Movie 3. 786-0 cells were transiently transfected with DHX9 siRNA #2. After 48h, the cells were infected with vMyx-GFP (MOI = 0.01) for 1h and replaced with fresh media. After 24h live images were captured using Evos microscope.

Supplemental Movie 4. 786-0 cells were transiently transfected with DHX9 siRNA #3. After 48h, the cells were infected with vMyx-GFP (MOI = 0.01) for 1h and replaced with fresh media. After 24h live images were captured using Evos microscope.

Supplemental Movie 5. 786-0 cells were transiently transfected with DHX9 siRNA #4. After 48h, the cells were infected with vMyx-GFP (MOI = 0.01) for 1h and replaced with fresh media. After 24h live images were captured using Evos microscope.
